# Metabolism and prebiotics activity of anthocyanins from black rice (*Oryza sativa* L.) *in vitro*

**DOI:** 10.1371/journal.pone.0195754

**Published:** 2018-04-09

**Authors:** Yongsheng Zhu, Hanju Sun, Shudong He, Qiuyan Lou, Min Yu, Mingming Tang, Lijun Tu

**Affiliations:** 1 School of Food Science and Engineering, Hefei University of Technology, Hefei, Anhui, PR China; 2 School of Food Science and Technology, National Engineering Research Center of Seafood, Dalian Polytechnic University, Dalian, Liaoning, PR China; College of Agricultural Sciences, UNITED STATES

## Abstract

Anthocyanins are naturally active substances. In this study, anthocyanins from black rice were obtained by membrane filtration and column chromatography separation. Five anthocyanin monomers in black rice extract were identified by HPLC-MS/MS, and the major anthocyanin monomer (cyanidin-3-glucoside, C3G) was purified by preparative HPLC (Pre-HPLC). The proliferative effects of the anthocyanins on Bifidobacteria and Lactobacillus were investigated by determining the media pH, bacterial populations and metabolic products. After anaerobic incubation at 37 °C for 48 h, not only the pH of the media containing C3G was lower than that of the extract of black rice anthocyanin (BRAE), but the numbers of both Bifidobacteria and Lactobacillus were also significantly increased. Furthermore, hydroxyphenylpropionic, hydroxyphenylacetic, and hydroxybenzoic acids and other metabolites were detected by GC-MS *in vitro*. Our results revealed that the anthocyanins and anthocyanin monomers from black rice had prebiotic activity and they were metabolized into several small molecules by Bifidobacteria and Lactobacillus.

## Introduction

In recent decades, a wide range of chemosynthetic pigments have been used in foods and cosmetics because of their lower costs and good stability. However, the toxicity of chemosynthetic pigments is of great concern, which may lead to various disease [1]. Due to the pursuit of health, more and more people pay attention to the applications of natural pigments, and approximately 15 natural pigments, such as anthocyanins, betanins, carotenoids and chlorophyllin, have been officially authorized for use in the food industry [2].

Anthocyanins belong to flavonoids and possess the typical chemical structure of polyphenols. As important water-soluble natural pigments, anthocyanins are widespread in vegetables and fruits as well as responsible for the vivid red, purple and blue colors of flowers, plants and cereals [3]. There are no less than 500 anthocyanins in nature, and only six common structures, including cyanidin, peonidin, pelargonidin, petunidin, delphinidin and malvidin, have been found so far [4].

Black rice is a characteristic crop from gramineous plant rice with a long cultivation time. More than 60% of the world’s black rice is cultivated in China and other Asian countries [5]. It is interesting to mention that there are abundant anthocyanins located in the aleurone layer of black rice [6]. Recently, two kinds of anthocyanin monomers from Thai black rice (Khao Hom Nin) were identified by LC-ESI-MS/MS, and cyanidin-3-glucoside (C3G) was confirmed as the major individual anthocyanin [7]. Three types of anthocyanins, including C3G, peonidin-3-glucoside, and malvidin, were characterized from two Japanese black rice genotypes (Asamurasaki and Okunomurasaki) by HPLC, and a fourth anthocyanin (petunidin-3-glucoside) was newly discovered in a variety of Chinakuromai [8]. Nevertheless, there is little comprehensive information on the identification and purification of anthocyanins from black rice (*Oryza sativa L*.) cultured in southern China. Furthermore, the functional significance of anthocyanins from black rice has not been well documented yet.

Anthocyanins have been considered health-promoting substances because of their diverse healthy functions, such as antioxidant, anti-inflammatory, anti-cancerous and anti-proliferative activities, and their most attractive biological feature might owe to their capacity to affect the growth of vulnerable micro-organisms [9–11]. Several *in vitro* studies revealed that the numbers of potential beneficial bacteria (Bifidobacteria and Lactobacilli) increased after the consumption of purple sweet potato anthocyanins and grape seed extract [12, 13]. As a kind of polyphenol, anthocyanins are hardly absorbed in the small intestine and might be transformed into small molecular phenolic acids by gut microbiota through ring cleavage, dehydroxylation and methylation reactions. The metabolites generated from polyphenols have proven that polyphenols could selectively motivate the growth of beneficial bacterial and inhibit the proliferation of harmful bacteria [14, 15] However, information about the anthocyanins from black rice were co-cultured with specifically single bifidobacteria or lactobacilli and their *in vitro* effects on Bifidobacteria and Lactobacilli is scarce, and the potential pathways in the catabolism of the anthocyanins by Bifidobacteria and Lactobacilli are still unclear.

Therefore, the objective of the present study was to characterize anthocyanins from black rice and evaluate the modulatory effects of C3G on Bifidobacterium (*B*. *bifidum*, *B*. *adolescents* and *B*. *infantis*) and Lactobacillus (*L*. *acidophilus*) by *in vitro* fermentation. GC-MS was utilized to detect metabolites from the biotransformation of C3G, and the metabolic pathway was further discussed.

## Materials and methods

### Materials

Black rice (*Oryza sativa L*.) was purchased from the local market (No. 100 Maanshan South Road, Baohe District, Hefei City, China) and cultured in a suburb of Hefei city, Anhui Province, China. *Bifidobacterium bifidum* (CICC 6071), *Bifidobacterium adolescentis* (CICC 6070), *Bifidobacterium infantis* (CICC 6069) and *Lactobacillus acidophilus* (CICC 6096) were purchased from the Chinese Collection Center of Industrial Culture (Beijing, China). Methanol (HPLC-grade) and acetonitrile (HPLC-grade) were obtained from Tedia Co. Lnc., (Shanghai, China). Formic acid (88%) was purchased from Shanghai Chemical Reagents Co., Ltd. (Shanghai, China). The BS basal nutrient medium (pH 7.2) was purchased from Hope Bio-technology Co., Ltd. (Qingdao, China), and MRS basal nutrient medium (pH 5.7) was from Huankai Microbial Sci. & Tech. Co., Ltd. (Guangdong, China), respectively. A Cyanidin-3-glucoside standard was obtained from Zhongkezhijian Biotechnology Co., Ltd. (Beijing, China), and BSTFA (N,O-bis (trimethylsilyl) trifluoroacetamide) with 1% TMCS (trimethylchlorosilane) was purchased from the Aladdin biochemical technology Co., Ltd. (Shanghai, China). Milli-Q water was used through out the experiments.

### Extraction of black rice anthocyanin

BRAE (extract of black rice anthocyanin) was prepared according to our previous study [1]. Briefly, black rice powder was extracted three times with buffer solutions (prepared by 0.2 M disodium hydrogen phosphate and 0.1 M citric acid, pH 3.2) at a solid-liquid ratio of 1:12 for 80 min at 50 °C. The solutions were first filtered with a 3 kDa membrane at room temperature under an operation pressure of 0.5 MPa, and the filtrate was then loaded on a glass column (20 mm × 1000 mm) of AB-8 resin, and 80% (v:v) ethanol was subsequently used as the eluent at a flow rate of 1.0 mL/min. The eluate was collected and freeze-dried for further experiments.

### Identification of anthocyanins in BRAE

The identification of anthocyanins in BRAE was performed by an Agilent 1260 UPLC system (Agilent, USA) coupled with electrospray ionization tandem mass spectrometry (LC-ESI-MS/MS, Agilent, USA). The diode array detector was set at 520 nm. The anthocyanins were separated by an Agilent Eclipse Plus C_18_ (2.1 mm × 50 mm) with an average particle size of 1.8 μm at 0.2 mL/min at room temperature. The mobile phase consisted of A (water: formic acid = 99:1, v:v) and B (acetonitrile: formic acid = 99:1, v:v), with the following gradient elutions: 0–10 min, 95% A; 10–15 min, 95–5% A; 15–17 min, 5% A; 17–17.1 min, 5–95% A; and 17.1–20 min, 95% A. Mass analysis was performed on a Bruker Impact Ⅱ Q-TOF mass spectrometer (Bruker Co. Ltd., USA) in positive mode with a scanning interval of 200–1000 m/z. Nebulization was conducted at 310 °C, aided by concurrent N_2_ flow at 11.0 psi. Capillary and cone voltages were set at 3.5 kV and 40 V, while the drying gas flow rate was 5 L/min at 10 psi. The data were processed with Bruker Daltonics Smart Formula software (Bruker Co. Ltd., USA).

### Purification of C3G in BRAE

C3G was separated by preparative HPLC (Pre-HPLC, Lisure Co. Ltd., China) equipped with a column of C18 (25 mm × 200 mm, 10 μm, Galak Chromatography Technology Co. Ltd., China). A gradient elution composed of solvent A (water: formic acid = 90:10, v:v) and B (acetonitrile: methyl alcohol: formic acid: water = 25:25:10:40, v:v:v:v) was applied at a flow rate of 12 mL/min as follows: 0 min, 16% B; 8 min, 20% B; 11 min, 38% B; 26 min, 46% B; 27 min, 85% B; 34 min, 85% B; 35 min, 16% B; and 40 min, 16% B. BRAE was dissolved with solvents A and B (84:16, v/v) at concentration of 5 mg/mL, and the injection volume was 5 mL. Elution was performed at 520 nm with UV-vis detection, and the highest peak was collected at 17.2 ± 0.5 min. The HPLC and mass spectrometer were subsequently applied to verify the purity of C3G.

### Effect of C3G on the proliferation of Bifidobacteria and Lactobacilli

#### *In vitro* fermentation of C3G

The C3G was mixed with autoclaved nutrient basal medium (100 mL) at the concentrations of 0, 0.5, 1, 1.5, 2 and 2.5 mg/mL and then inoculated with 150 μL of bacterial slurry (0.15%, w/v) with a manual homogenizer. Subsequently, fermentation was performed in an anaerobic incubator (YQX-Ⅲ, Shanghai Wan Rui Industrial Co. Ltd., China) with an atmosphere of 10% H_2_, 10% CO_2_ and 80% N_2_ at 37 °C.

#### Measurement of media pH value

A precise pH meter (Shanghai Hong Yi Instrument Corp., Ltd, China) was used to measure the medium pH after a 48-h fermentation in anaerobic conditions at 37 °C.

#### Analysis of bacteria number

The dilution coating plate method was utilized to evaluate the bacterial number. Fifteen millilitres of medium was first poured into a sterilized culture dish, followed by 200 μL of activated bacterial dilution (10 times dilution), which was poured into the plate and spread evenly with a coating bar. After anaerobic culture at 37 °C for 48 h, the colony numbers were counted.

#### GC-MS analysis

The broth samples were repetitively collected after 1, 2, 4, 24 and 48 h of incubation in 1.5 mg/mL treatment groups. After centrifugation at 10000 r/min at 4 °C for 10 min, one hundred and fifty microliters of fermentation broth and 50 μL of internal standard (5 μg/mL 4-amino salicylic acid) were extracted with 500 μL of ethyl acetate. The supernatant was dried under a nitrogen stream after centrifugation at 12000 r/min for 15 min. Then, the dried residue was mixed with 30 μL of BSTFA (with 1% TMCS) and derivatized at 70 °C for 60 min.

An Agilent 7890A gas chromatography system coupled with an Agilent 5975C inert MSD system (Agilent Technologies Inc., CA, USA) was used to analyse the metabolites. Separation was performed with an Rxi-5 Sil capillary column (30 m × 0.25 mm × 0.25 μm, Restek corporation, Bellefonte, PA, USA). Helium was used as a carrier gas at a flow rate of 1 mL/min. The injection volume was 1 μL in splitless mode, and the solvent delay time was 6 min. The initial oven temperature was held at 70 °C for 2 min, ramped up to 160 °C at a rate of 6 °C/min, to 240 °C at a rate of 10 °C/min, and to 300 °C at a rate of 20 °C/min and finally held for 6 min. The temperatures of the injector, transfer line, and electron impact ion source were set at 250, 250, and 230 °C, respectively. The electron energy was 70 eV, and data were collected in full scan mode (m/z 50–600). The experimental data was analysed by MSD ChemStation (version E.02.02.1431, Agilent Technologies, Inc.). The metabolite content was presented as the relative content and calculated by RC = A_m_/A_IS_, where RC represents the relative content of the metabolite, A_m_ represents the peak area of the metabolite, and A_IS_ represents the peak area of IS.

### Statistical analysis

Significant differences (*P < 0*.*05*) between the experimental groups were evaluated by ANOVA (one-way analysis of variance) using SPSS software for Windows (Version 17, IBM Corp., USA).

## Results

### Identification of anthocyanins from black rice

**Fig 1:**
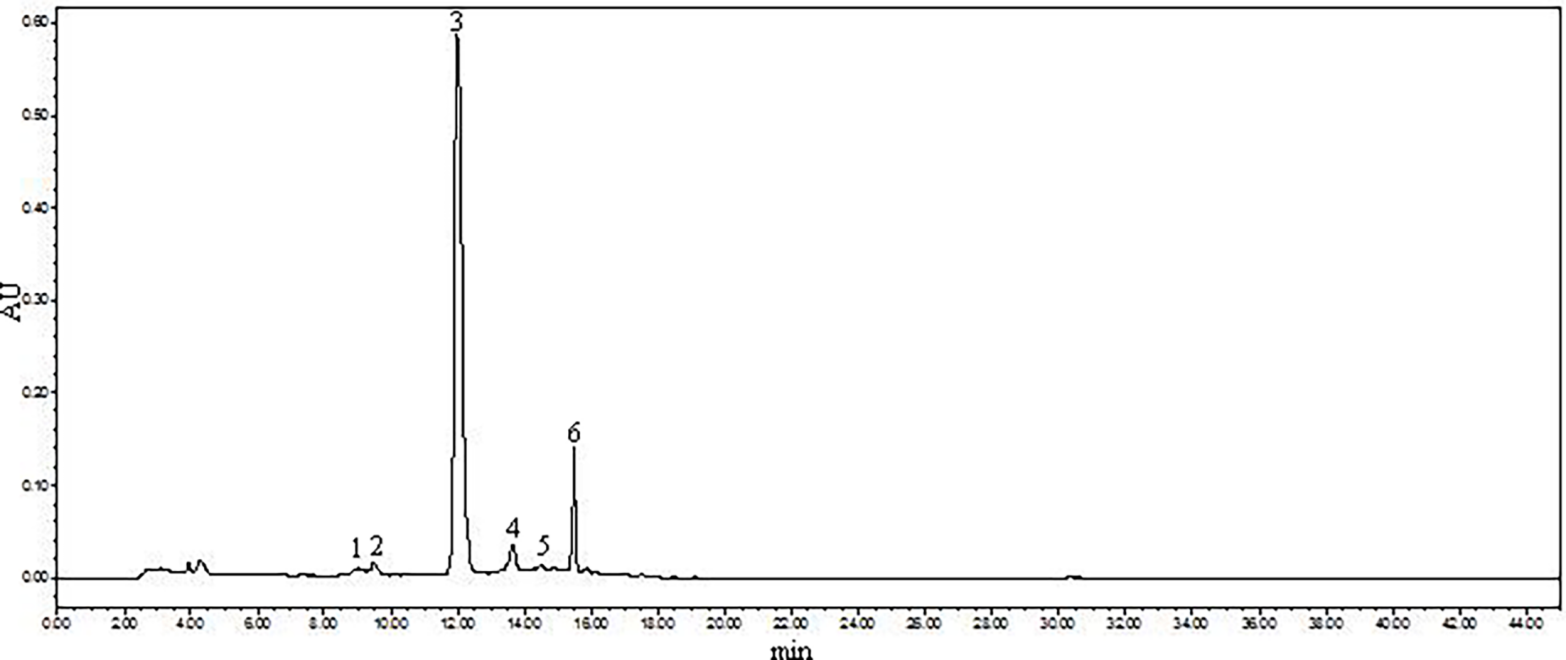
HPLC chromatogram of the black rice extract detected at 520 nm. Peak 1, cyanidin-3,5-diglucoside; Peak 2, not identified; Peak 3, cyanidin-3-glucoside; Peak 4, cyanidin-3-rutinoside; Peak 5, peonidin-3-glucoside; and Peak 6, peonidin-3-rutinoside.

**Fig 2:**
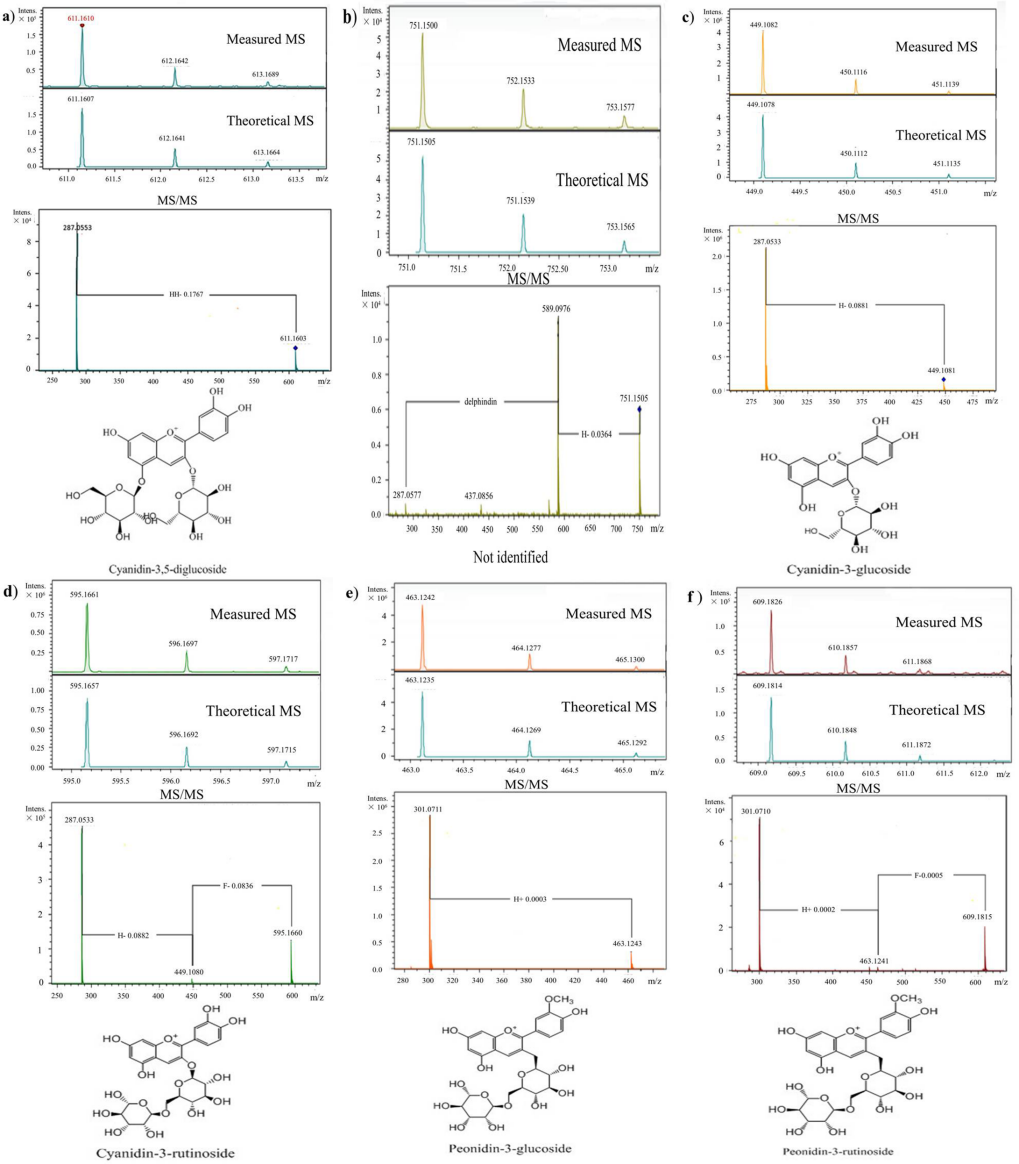
MS, MS/MS spectra of the six peaks in the HPLC analysis and the chemical structure of the anthocyanin monomers detected in the black rice extract. a (Peak 1), cyanidin-3,5-diglucoside; b (peak 2), not identified; c (peak 3), cyanidin-3-glucoside; d (peak 4), cyanidin-3-rutinoside; e (peak 5), peonidin-3-glucoside; and f (peak 6), peonidin-3-rutinoside.

As shown in Fig 1, six peaks were obtained, and peak 3 was calculated as the main component because of the 86.98% proportion in the extract of black rice anthocyanins. Then, each peak was characterized by HPLC-ESI-MS/MS, and the MS/MS spectra and chemical structures of the anthocyanins are depicted in Fig 2.

As presented in Fig 2A, peak 1 possessed a molecular ion [M]^+^ at m/z 611 and a fragment ion at m/z 287 [M-162-162]^+^. Since the theoretical data of cyanidin-3,5-diglucoside was m/z 611, the loss of 162 from the molecular ions revealed the existence of a glucose moiety in the structures of the identified peak, and the measured m/z data (m/z 611) of peak 1 were identified as cyanidin-3,5-diglucoside. The MS spectrum of peak 2 (Fig 2B) was presented as a molecular ion [M]^+^ at m/z 751 and two fragment ions at m/z 589 ([M-162]^+^) as well as 287 ([M-162-302]^+^). The losses of 162 and 302 were responsible for the one glucose moiety and one delphinidin moiety, respectively, and a fragment ion at m/z 287 corresponded to the cyanidin moiety. Unfortunately, peak 2 was not identified because information for both the delphinidin and cyanidin was found in the MS profile. As shown in Fig 2C, a molecular ion [M]^+^ at m/z 449 was observed in the MS spectrum of peak 3, and the molecular ion could be fragmented into one ion at m/z 287 [M-162]^+^, indicating the loss of one glucose moiety. Then, peak 3 was confirmed as cyanidin-3-glucoside. According to the analysis of the measured MS data and the comparison with the theoretical data, peak 4 (Fig 2D) could be identified as cyanidin-3-rutinoside. As the molecular ion at m/z 463 and the characteristic fragmentation pattern of the molecular ion at m/z 301 [M-162]^+^ were found in peak 5 (Fig 2E), the corresponding component was identified as peonidin-3-glucoside. The MS profile of peak 6 (Fig 2F) exhibited a molecular ion [M]^+^ at m/z 609 and a primary fragment ion at m/z 301 [M-308]^+^, which corresponded to the molecular ion loss of one rutinoside moiety. Hence, peak 6 was tentatively identified as peonidin-3-rutinoside.

### C3G purification by Pre-HPLC

**Fig 3:**
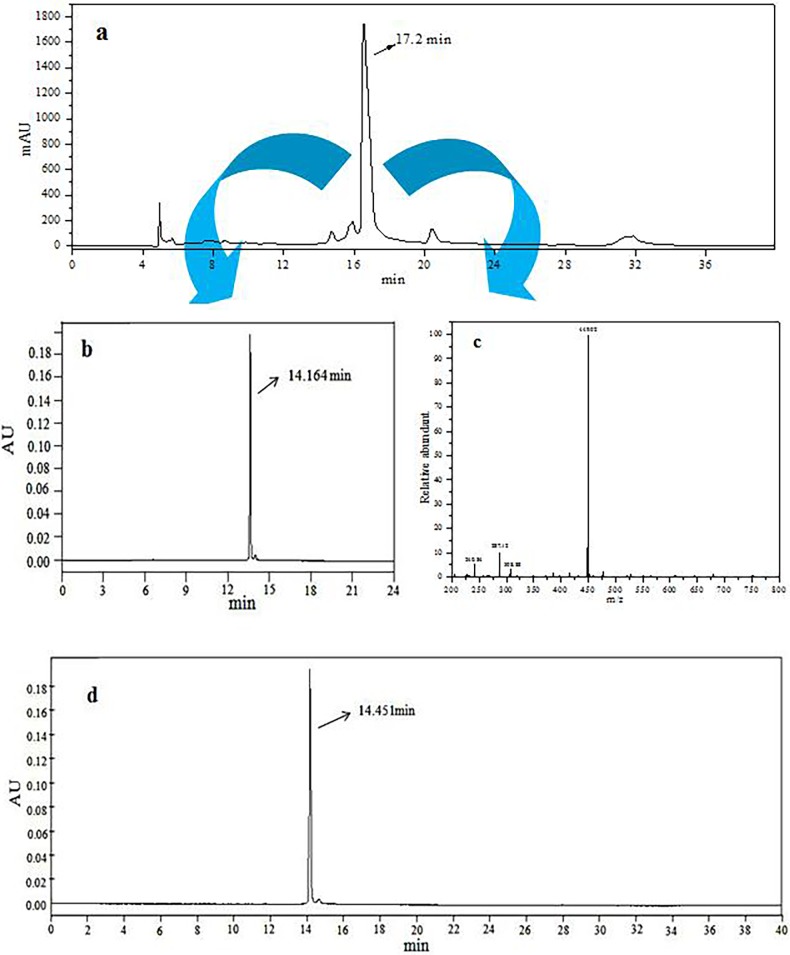
Purification and verification of C3G. (a) Pre-HPLC chromatogram of black rice anthocyanin extract; (b) HPLC chromatogram of C3G purified by Pre-HPLC; (c) MS spectrum of C3G purified by Pre-HPLC; (d) HPLC chromatogram of C3G standard.

Pre-HPLC was further applied to purify the main anthocyanin monomer (C3G). As shown in Fig 3A, the peak at a retention time of 4.5 min was confirmed as the solvent peak, while the peak at a retention time of 17.2 min was the major peak, which was subsequently collected. Based on the HPLC analysis (Fig 3B), the collection was initially identified as C3G, with a retention time of 14.164 min, which was similar to that of the standard (Fig 3D, 14.451 min), and the purity could be defined at no less than 98% by the peak integration. Furthermore, the relative molecular mass of m/z 449 obtained by LC-MS analysis (Fig 3C) confirmed the efficiency of the pre-HPLC for C3G purification.

### The pH values of bacterial media after C3G and BRAE incubations

**Fig 4:**
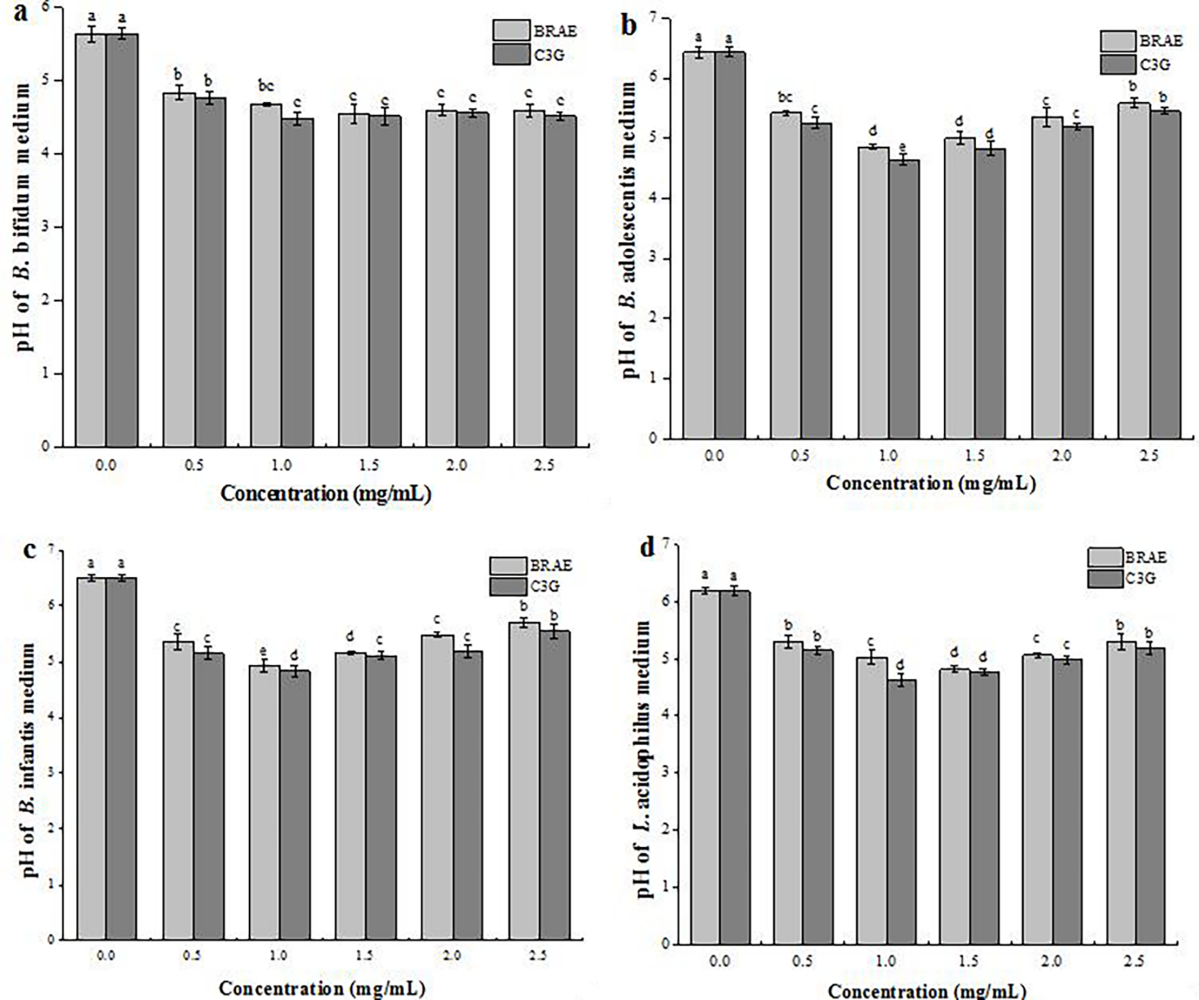
Effect of BRAE and C3G on the pH of diverse bacterial media under anaerobic conditions at 37 °C for 48 h. (a) *B*. *bifidum*; (b) *B*. *adolescentis*; (c) *B*. *infantis*; (d) *L*. *acidophilus*. Data were obtained from three independent experiments and represent the mean values and SD. Different lowercase letters indicate significant differences (*P < 0*.*05*) in the media pH values with different concentrations of the same microorganism.

A concentration-dependent analysis was conducted to study the effects of anthocyanins on the pH of the bacterial medium. C3G and BRAE were co-cultured with Bifidobacteria and Lactobacillus under anaerobic conditions at 37 °C for 48 h, and the results are shown in Fig 4. As shown in Fig 4A, the pH of the *B*. *bifidum* media initially decreased from 5.63 to 4.48 with an increasing concentration of C3G and reached lowest pH (pH 4.48) at a concentration of 1.0 mg/mL. Then, the pH remained at around 4.55 as it extended from 1.0 to 2.0 mg/mL and decreased to 4.50 at 2.5 mg/mL. The pH of the *B*. *adolescentis* media decreased from 6.44 to 4.63, and the lowest pH (pH 4.64) was found at 1.0 mg/mL in C3G addition, followed by a increase from 4.83 to 5.45 in the medium pH as the C3G concentration range from 0 to 2.5 mg/mL (Fig 4B). As shown in Fig 4C and 4D, the pH values of the *B*. *infantis* and *L*. *acidophilus* media were similar to that of *B*. *adolescentis*, and the minimum pH values were at 4.83 and 4.62, respectively. Meanwhile, a significant decrease in the pH was also detected in medium containing BRAE after incubation for 48 h (*P < 0*.*05*), and the trend coincided with that of C3G. The lowest pH values were in the following order: *B*. *infantis* (pH 4.92) > *B*. *adolescentis* (pH 4.86) > *L*. *acidophilus* (pH 4.81) > *B*. *bifidum* (pH 4.54) in media containing BRAE at a concentration range of 1.0–1.5 mg/mL. All the results indicated that the pH of the media containing C3G was lower than that of BRAE, indicating the high potential efficiency of the anthocyanin monomer.

### Effects of C3G and BRAE on bacterial proliferation

The effects of the anthocyanins on bacterial proliferation were further assayed, and the results are shown in Table 1. Compared with the control, C3G clearly induced significant increases (*P* < 0.05) in the numbers of Bifidobacteria and Lactobacilli, except for *B*. *adolescentis* treated by 2.5 mg/mL C3G. The numbers of *Bifidobacteria* (*B*. *adolescentis*, *B*. *infantis*, *B*. *bifidum*) gradually increased with increasing concentrations of the anthocyanins, and the reduction in the growth rate was subsequently found as the concentration further increased. At 1.0 mg/mL, C3G and BRAE showed the best proliferative effect on *Bifidobacteria* after cultivation for 48 h. The maximum populations of *L*. *acidophilus* appeared at a concentration of 1.5 mg/mL in broth containing BRAE after incubation for 48 h, while, C3G exerted the best growth-promotion on *lactobacilli* at concentrations of 1.0 and 1.5 mg/mL. The proliferation effect decreased in the following order: *B*. *bifidum* (9.13 cfu/mL) > *B*. *adolescentis* (8.15 cfu/mL) > *B*. *infantis* (8.13 cfu/mL*)* > *L*. *acidophilus* (8.10 cfu/mL) in media containing C3G at an optimal concentration. *B*. *bifidum*, in particular, seemed quite robust, and the numbers of *B*. *bifidum* were more than the other three strains as described above, and significant differences were obtained in the C3G and BRAE treated.

**Table 1:** Numbers (log_10_ cell/mL) of Bifidobacteria and Lactobacilli groups in anaerobic fermentation broth containing C3G and BRAE at 37 °C for 48 h.

Samples	Concentrations(mg/mL)	Number of probiotics (log cfu/mL)			
		*B*. *infantis*	*B*. *adolescentis*	*B*. *bifidum*	*L*. *acidophilus*
**BRAEs**	0 (Control)	7.66±0.07^c^	7.74±0.05^b^	8.65±0.11^b^	7.65±0.09^c^
	0.5	7.89±0.14^ab^	7.91±0.15^ab^	8.91±0.09^a^	7.87±0.05^b^
	1.0	8.03±0.11^a^	8.07±0.16^a^	9.08±0.12^a^	7.98±0.04^ab^
	1.5	7.98±0.03^ab^	7.98±0.07^ab^	9.07±0.21^a^	8.07±0.11^a^
	2.0	7.82±0.05^abc^	7.85±0.12^ab^	9.06±0.17^a^	7.96±0.16^ab^
	2.5	7.79±0.21^bc^	7.79±0.18^b^	9.04±0.13^a^	7.87±0.08^b^
**C3G**	0 (Control)	7.66±0.13^d^	7.74±0.08^c^	8.65±0.08^c^	7.65±0.08^c^
	0.5	7.93±0.16^bc^	7.96±0.06^b^	8.95±0.09^b^	7.95±0.05^b^
	1.0	8.13±0.09^a^	8.15±0.14^a^	9.13±0.09^a^	8.10±0.02^a^
	1.5	8.07±0.07^ab^	8.02±0.04^ab^	9.08±0.12^ab^	8.10±0.05^a^
	2.0	7.94±0.07^abc^	7.90±0.09^b^	9.06±0.06^ab^	8.00±0.04^b^
	2.5	7.86±0.05^c^	7.86±0.06^bc^	9.05±0.05^ab^	7.92±0.05^b^

Data were obtained from three independent experiments and represented as the mean values ± SD. Different lowercase letters indicate significant differences (*P < 0*.*05*) in the numbers of probiotics among different concentrations of the same microorganism.

As shown in Table 1, increases in the bacterial counts were observed in all the tested substrates, and the changing tendency of bacterial counts in broth containing BRAE or C3G was similar to the alteration of the medium pH, while, C3G exerted better proliferative effects on human intestinal bacteria than BRAE at concentrations of 1.0 and 1.5 mg/mL.

### Metabolites of C3G by *in vitro* fermentation

**Fig 5:**
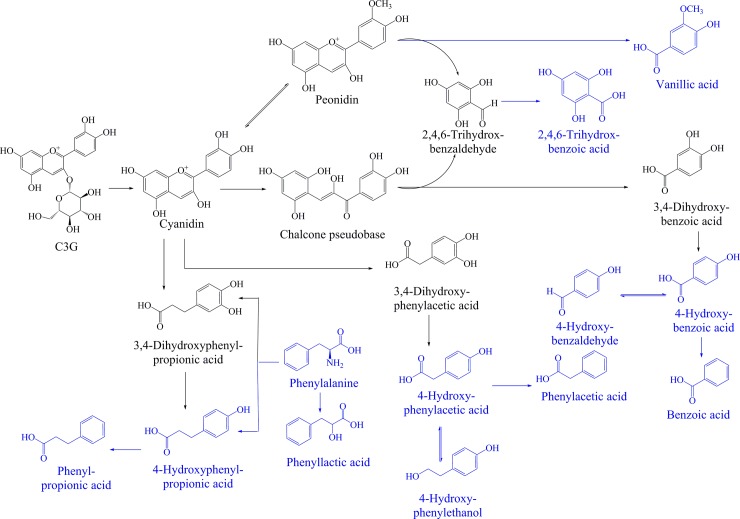
Proposed metabolic pathway of C3G based on the metabolites detected in the Bifidobacteria and Lactobacilli fermentation broth. Blue metabolites were detected, and grey metabolites were not detected in the GC-MS experiment.

The diverse phenolic acids and alcohols, including intermediate and final metabolites, were detected in the media containing C3G (Table 2), and the variations in the relative contents were presented in Table 3. Furthermore, the metabolic pathway of C3G was analysed based on the enzyme catalysis and cleavage of the basic skeleton of anthocyanins (Fig 5).

**Table 2:** C3G metabolites produced by Bifidobacteria and Lactobacilli through GC-MS after BSTFA derivatization.

No.	Retention time (min)	Compound	Molecular ions (m/z)
**1**	11.26	benzoic acid	179
**2**	12.39	phenylacetic acid	193
**3**	15.00	phenylpropanoic acid	104
**4**	16.18	2,4,6-trihydroxybenzoic acid	179
**5**	16.59	4-hydroxybenzaldehyde	223
**6**	17.94	phenylalanine	174
**7**	18.07	4-hydroxyphenylethanol	179
**8**	18.26	phenyllactic acid	193
**9**	19.01	4-hydroxybenzoic acid	267
**10**	19.18	4-hydroxyphenylacetic acid	179
**11**	20.89	4-hydroxyphenylpropionic acid	179
**12**	20.93	4-hydroxy-3-methoxybenzoic acid	297

**Table 3:** Relative content of C3G metabolites produced by Bifidobacteria and Lactobacilli at different incubation times in anaerobic conditions at 37 °C.

Compound (relative content)	*B*. *infantis*					*B*. *bifidum*					*B*. *adolescentis*					*L*. *acidophilus*				
	Incubation time (h)																			
	1	2	4	24	48	1	2	4	24	48	1	2	4	24	48	1	2	4	24	48
benzoic acid	0.42	0.73	1.23	1.04	1.51	0.97	1.16	1.20	1.26	1.81	0.57	0.89	1.16	1.47	1.35	0.45	0.84	1.33	1.65	1.14
phenylacetic acid	0.02	0.01	0.01	0.02	0.03	0.02	0.03	n.d	0.01	0.03	n.d	0.02	0.02	0.01	0.01	n.d	n.d	0.02	0.01	0.02
phenylpropanoic acid	0.07	0.09	0.15	0.09	0.12	0.09	0.09	0.11	0.12	0.08	0.05	0.07	0.09	0.14	0.05	0.17	0.19	0.28	0.43	0.29
2,4,6-trihydroxybenzoic acid	0.01	0.02	0.04	0.01	n.d	0.03	0.03	0.04	0.04	0.05	n.d	0.02	0.03	0.05	0.01	0.02	0.04	0.07	0.03	0.05
4-hydroxybenzaldehyde	0.02	0.06	0.12	0.06	0.04	0.05	0.07	0.11	0.12	0.03	0.04	0.05	0.08	0.16	0.09	0.03	0.05	0.05	0.08	0.05
phenylalanine	n.d	0.05	0.20	0.02	0.03	0.01	0.01	0.08	0.11	0.07	0.03	0.05	0.06	0.02	0.03	0.07	0.05	0.07	0.09	0.13
4-hydroxyphenylethanol	0.05	0.06	0.12	0.05	0.04	0.06	0.07	0.08	0.06	0.06	0.02	0.04	0.05	0.16	0.06	0.08	0.12	0.15	0.19	0.18
phenyllactic acid	0.30	0.60	7.98	9.63	11.30	0.42	0.43	2.44	3.76	2.64	3.84	4.52	7.61	16.67	10.74	1.42	1.89	0.70	12.62	14.10
4-hydroxybenzoic acid	0.03	0.04	0.17	0.06	0.02	0.10	0.14	0.16	0.08	0.02	0.06	0.05	0.12	0.16	0.15	0.04	0.05	0.07	0.12	0.07
4-hydroxyphenylacetic acid	0.27	0.24	0.05	0.09	0.05	0.14	0.16	0.06	0.04	0.04	0.07	0.06	0.13	0.05	0.06	0.08	0.05	0.06	0.17	0.05
4-hydroxyphenylpropionic acid	0.08	0.10	0.15	0.11	0.15	0.10	0.11	0.14	0.16	0.09	0.06	0.09	0.10	0.15	0.26	0.03	0.07	0.17	0.24	0.18
4-hydroxy-3-methoxybenzoic acid	n.d	0.02	0.07	0.08	0.03	0.09	0.10	0.08	0.05	0.02	0.04	0.13	0.15	0.12	0.17	n.d	n.d	0.04	0.05	0.03

n.d, not detected.

Bacterial media were taken from the *in vitro* fermentation at 1, 2, 4, 24 and 48 h, and twelve metabolites of C3G were detected in all bacterial media by GC-MS.

As shown in Table 3, a wide range of metabolites was found in the fermentation medium, including 4-hydroxyphenylethanol, 4-hydroxybenzaldehyde, phenyllactic acid, benzoic acid, phenylacetic acid, phenylpropanoic acid and three hydroxylated forms of the last three substances. After incubation with *B*. *infantis*, C3G led to a significant increase in the relative content of phenyllactic acid, and the highest content of phenyllactic acid was obtained at 48 h. Phenyllactic acid was the main metabolic product caused by the bacterial degradation of C3G, and it was also obtained in the medium of the other three strains after incubation for 48 h. Benzoic acid was the second main fermentation product in the media of *B*. *infantis* and *B*. *bifidum*, and it reached up to a maximum level at 24 h in the media of *B*. *adolescentis* and *L*. *acidophilus*. Phenylacetic acid was the lowest metabolite in the fermentation media of the four strains. Initially, the relative content of phenylacetic acid decreased and then remained stable followed by slow increase during fermentation. The phenylacetic acid in *B*. *bifidum* media and *B*. *adolescentis* media were not detected at 4 h and 1 h, respectively; while, phenylacetic acid was not obtained during the first 2 h cultivation in case of *L*. *acidophilus*. Apart from these representative metabolites, some other metabolites, such as 2,4,6-trihydroxybenzoic, 4-hydroxyphenylethanol, 4-hydroxybenzoic acid, 4-hydroxyphenylacetic acid and 3-methoxy-4-hydroxybenzoic acid were obtained during the metabolism of C3G with probiotics, which led to initial increase of content followed by decrease.

## Discussion

In our study, five kinds of individual anthocyanins from black rice (Oryza sativa L.) were identified as cyanidin-3,5-diglucoside, cyanidin-3-glucoside, cyanidin-3-rutinoside, peonidin-3-glucoside and peonidin-3-rutinoside, while, C3G was confirmed as the major anthocyanin. Our result was similar to previous studies for black rice planted in the north of China, such as Shanxi and Jilin provinces [5, 16] The diversity of anthocyanin monomers seemed to depend on the difference in the planting area for the black rice, as only two individual anthocyanins were characterized using LC-DAD-ESI/MS from ten Korea black rice varieties. Nevertheless, C3G was also the most principal anthocyanin, which was similar to our results [17]

As shown in the results, the decrease in the media pH (Fig 3) and increase in the population of bacteria (Table 1) reflected the modulatory effects of C3G and BRAE on Bifidobacteria and Lactobacilli. The latter is considered the predominant community of the intestinal microbiota in animals and humans, and some of them have been demonstrated as probiotics and are responsible for body health at different levels [18] The decrease in the media pH indicated the production of organic acids containing phenolic acid and short chain fatty acids, implying the consumption of anthocyanins by Bifidobacteria and Lactobacilli. It has been reported that purple sweet potato anthocyanins can be utilized by human intestinal bacteria, and a high amount of short chain fatty acids, such as formic acid, acetic acid, propionic acid, butyric and lactic acid, were generated [13]. On the other hand, C3G is connected by β-glucoside bonds with glucoside, and the probiotics could secrete the β-glucosidases for anthocyanin degradation, which resulted in more energy for the growth of the bacteria. Furthermore, as a basic skeleton of anthocyanidin, 2-phenylbenzopyrylium contains three benzene rings and it was further degraded into phenolic acid, as well as short-chain fatty acids after the breakdown of β-glucoside bonds, which led to the decrease in the media pH and provided a more suitable pH for the growth of probiotics [19]. These results suggested that black rice anthocyanins harbour remarkable prebiotic potential. To investigate the metabolism of black rice anthocyanins, GC-MS was used to determine the metabolites from the biotransformation of C3G by Bifidobacteria and Lactobacilli, and twelve metabolites with different relative contents were detected in the four strains of media (Table 2). During the period for the detection of metabolites, the compound 2,4,6-trihydroxybenzoic acid was detected which might result from the oxidation of 2,4,6-trihydroxybenzaldehyde, and the degradation of the chalcone pseudobase led to the presence of 2,4,6-trihydroxybenzaldehyde [20]. Unfortunately, the chalcone pseudobase and 2,4,6-trihydroxybenzaldehyde were not detected in our study. The formation of phenyllactic acid was observed during the incubation of C3G with probiotics. Phenyllactic acid was recognized for its inhibition of the fungus growth and pathogenetic bacteria, such as *Staphylococcus aureus* and *Escherichia coli* which was further confirmed by a significant decrease in the population of gastrointestinal *E*. *coli* in chickens by feeding them phenyllactic acid [21]. The appearance of phenyllactic acid was related to the existence of phenylalanine and tyrosine [21], and phenylalanine was detected in our study, but tyrosine was not found. According to a previous study, a variety of phenylpropionic acid derivatives could be involved in the metabolic pathway of phenylalanine [22]. During the formation of phenylpropionic acid, many reactions, including dehydroxylation, α-oxidation, β-oxidation and rearrangement reactions occurred. Thus, 3-(3,4-dihydroxyphenyl)-propionic acid, 3-(3-hydroxyphenyl)-propionic acid and 3,4-dihydroxyphenylacetic acid were not found in our study because of their minimal contents. But their further degradation products were observed in our study. Dehydroxylation at C-3 of 3-(3,4-dihydroxyphenyl)-propionic acid would lead to the appearance of 4-hydroxyphenylpropionic acid. Similarly, the metabolic product (4-hydroxyphenylacetic acid) arose from the dehydroxylation at C-3 of 3,4-dihydroxyphenylacetic acid. It should be noted that 4-hydroxyphenylethanol was detected in the fermentation medium, and it was probably produced from the reduction reaction of 4-hydroxyphenylacetic acid.

Vanillic acid (4-hydroxy-3-methoxybenzoic acid) was detected in bacterial metabolites, which might have a variety of biological activities, such as antihyperlipidaemic, free radical scavenging activity and inhibition of hepatic fibrosis in chronic liver injury [23, 24]. An interconversion between cyanidin and peonidin possibly occurred during fermentation progress. Vanillic acid was thought to arise from the degradation of peonidin [25], and its alternative pathway was the methylation of 3,4-dihydroxybenzoic acid by catechol-*O*-methyltransferase [26]. The compound 3,4-dihydroxybenzoic acid was detected in our preliminary experiments, but the content was below the limit of detection. At the end of metabolism, benzoic acid, phenylacetic acid, phenylpropanoic acid and 4-hydroxybenzoic acid were detected, suggesting the existence of the decarboxylation of corresponding precursor substances [27]. It has been reported that benzoic acids and 4-hydroxybenzoic acids are important intermediate products of hippuric acid and 4-hydroxyhippuric acid, respectively [22]. The formation of 4-hydroxybenzoic acid was obtained by decarboxylation of 3,4-dihydroxybenzoic acid, which was further transformed into benzoic acid by the same mechanism. The presence of these metabolites might refer to the activities of the β-glucosidase, β-glucuronidase and α-rhamnosidase of the gut flora [25]. Similar to 4-hydroxyphenylethanol, the presence of 4-hydroxybenzaldehyde might result from the reduction reaction of 4-hydroxybenzoic acid. As shown in Fig 5, the degradation of C3G was involved with numerous reactions, such as hydrolysis, dehydroxylation and methoxylation. At first, C3G might lose a glucoside because of the existence of β-glucosidase secreted from Bifidobacteria and Lactobacilli and was degraded into cyaniding [28]. Then, the heterocycle fission of cyanidin was carried out by Bifidobacteria and Lactobacilli, leading to the presence of a chalcone pseudobase [25]. Finally, cyanidin and chalcone pseudobases were further degraded into phenolic acids through oxidation, dehydroxylation, α-oxidation, β-oxidation, rearrangement reactions and methylation. Therefore, most of these metabolites were phenolic acids which resulted in direct decrease in the media pH and their further generation led to acidification of the medium environment which could promote the absorption of minerals [29]. Meanwhile, the proliferation of probiotics could be interpreted based on the above mentioned mechanisms. To summarize, the metabolic and prebiotic activities of black rice anthocyanins will help us to better understand the role of anthocyanins in regulating the intestinal environment.

## Conclusions

In conclusion, five anthocyanins were identified by HPLC-ESI-MS/MS, and C3G was obtained by preparative HPLC. The numbers of probiotics significantly increased after supplementation with anthocyanins, and the pH of the media was simultaneously decreased. An extensive range of metabolites, such as 2,4,6-trihydroxybenzoic acid, 4-hydroxybenzaldehyde, benzoic acid, phenylacetic acid, and phenylpropanoic acidwere found in the basal media containing C3G. A variety of phenolic acids were obtained after the bacterial metabolism of C3G. The modulatory effect of C3G on the intestinal microbiota by *in vivo* fermentation will provide a firm evidence of the health benefit from anthocyanin diet. More attention should be given for further exploration of underlying prebiotic mechanism of C3G through suitable animal models followed by clinical applications.
